# Intratumoral microbiota composition in women’s cancers: a systematic review and meta-analysis

**DOI:** 10.3389/fonc.2025.1544786

**Published:** 2025-06-12

**Authors:** Qin Wen, Shubin Wang, Shunlian Fu, Xinxiang Zhou, Yalan Min, Jinyi Lang, Meihua Chen

**Affiliations:** ^1^ School of Medicine, University of Electronic Science and Technology of China, Chengdu, China; ^2^ Department of Radiation Oncology, Radiation Oncology Key Laboratory of Sichuan Province, Sichuan Clinical Research Center for Cancer, Sichuan Cancer Center, Sichuan Cancer Hospital & Institute, Affiliated Cancer Hospital of University of Electronic Science and Technology of China, Chengdu, China; ^3^ School of Clinical Medicine, Chengdu University of Traditional Chinese Medicine, Chengdu, China

**Keywords:** intratumoral microbiota, 16s rrna gene sequencing, breast cancer, gynecologic cancer, estrogen, meta-analysis

## Abstract

**Background:**

The intratumoral microbiota has attracted considerable interest in carcinogenesis, progression, and treatment owing to advancements in sequencing technology. This systematic review provides a comprehensive overview of the current literature regarding the diversity and compositional characteristics of the intratumoral microbiota in women’s cancers. Additionally, it also explores potential associations among intratumoral microbiota, estrogen, and anti-tumor therapies.

**Methods:**

A comprehensive literature search was conducted using PubMed, Embase, Web of Science, and the Cochrane Library from their inception to May 1, 2024. The review protocol was pre-registered in PROSPERO (CRD 42024601213). Articles were assessed utilizing the Newcastle-Ottawa Scale (NOS). To estimate the effect size and variability in microbial diversity changes, the standardized mean difference (SMD) and 95% confidence intervals (CIs) were employed. The systematic review adhered to PRISMA reporting guidelines, and meta-analyses were performed using Review Manager version 5.4.

**Results:**

This systematic review included 29 of 8,291 studies after a thorough screening process. Of the 22 studies investigating α-diversity in women’s cancers, disease-free controls, and those with benign conditions, notable changes in diversity indices were observed. Compared to adjacent normal tissues, the Simpson index significantly decreased in breast cancer (SMD = -0.75, 95% CI: [-0.94, -0.55]) and endometrial cancer (SMD = -0.83, 95% CI: [-1.37, -0.28]). The Chao1 index was reduced in endometrial cancer tumor tissues relative to normal tissues (SMD = -2.25, 95% CI: [-3.13, -1.36]), while the Shannon index decreased in ovarian cancer tumor tissues (SMD = -0.61, 95% CI: [-1.18, -0.04]). In comparisons between tumor and benign tissues, the Chao1 index was decreased (SMD = -0.64, 95% CI: [-1.20, -0.08], I² = 0%), while the Simpson index was increased (SMD = 0.36, 95% CI: [0.01, 0.71], I² = 0%) in patients with ovarian cancer. Other microbial diversity indices showed no significant differences between tumor and non-tumor tissues. At the phylum level, *Fusobacteriota* were enriched in tumor tissues, while *Firmicutes* and *Actinobacteria* predominated in non-tumor tissues. At the genus level, *Pseudomonas*, *Porphyromonas*, *Atopobium*, *Peptoniphilus*, and *Acinetobacter* were consistently more abundant in cancerous tissues. Microbial alterations were also linked to estrogen receptor (ER) status, with *Alkanindiges* negatively correlated with ER status in two studies. Furthermore, one study on the effect of antineoplastic therapy indicated that neoadjuvant chemotherapy reduced microbial diversity in breast cancer patients (n = 15 vs. n = 18) (Shannon index: SMD = -0.95, 95% CI: [-1.68, -0.22]).

**Conclusion:**

This study highlights significant differences in microbiota composition between tumor and non-tumor tissues in women’s cancers, emphasizing changes in intratumoral microbiota influenced by estrogen and antineoplastic treatments. Further research is needed to explore the potential for developing targeted therapies based on estrogen-driven microbiota alterations. Investigations may yield insights into the enhancement of female reproductive health and the improvement of treatment efficacy for female cancers.

**Systematic review registration:**

https://www.crd.york.ac.uk/PROSPERO/view/CRD42024601213, identifier CRD 42024601213.

## 1 Introduction

The human microbiota, comprising a diverse community of microorganisms—including bacteria, fungi, viruses, and archaea—resides across various body sites and plays a crucial role in human health and disease. In particular, accumulating evidence has highlighted its significant involvement in cancer initiation, progression, and prognosis (1). Currently, considerable attention has been devoted to the gut, skin, oral, and vaginal microbiota due to their interactions with the host and therapeutic implications. With the discovery of the microbiota within tumor tissues, researchers are increasingly investing in the interaction between the intratumoral microbiota and cancer. Discoveries have identified microbiota within tumor tissues, which has sparked increasing interest in elucidating the interactions between the intratumoral microbiota and cancer. Mounting evidence indicates that the intratumoral microbiota may influence tumor development through several mechanisms, including DNA mutations, activation of oncogenic pathways, and promotion of chronic inflammation (2, 3). Additionally, intratumoral bacteria may modulate antitumor immunity by activating immune cells, triggering the STING signaling pathway, promoting the maturation of tertiary lymphoid structures (TLS), and presenting microbiota-derived antigens, thereby influencing cancer progression (4, 5).

Among women cancers—namely, breast, ovarian, cervical, and endometrial cancers—the role of the microbiota in tumor development has garnered increasing recognition. Certain bacterial taxa, such as *Firmicutes* and *Proteobacteria*, have been associated with immune escape and chemoresistance in breast cancer (BC) (6, 7). In ovarian cancer (OC), the intratumoral microbiota may also play a role in tumorigenesis by modulating hormone metabolism. In endometrial cancer (EC), the relative abundance of *Micrococcus* has been positively correlated with elevated levels of inflammatory cytokines, including IL-6 and IL-17 (8, 9). Furthermore, in cervical cancer (CC), *Lactobacillus iners* has been shown to induce resistance to chemotherapy and radiotherapy, potentially through lactic acid-mediated metabolic alterations (10).

Estrogen, a key sex hormone in women, plays a central role in regulating cell proliferation, differentiation, and apoptosis through its binding to intracellular estrogen receptors. Dysregulations in estrogen levels, metabolism, and receptor expression are implicated in cancer development. Notably, complex interactions between estrogen and the microbiota have emerged as critical factors in the pathophysiology of hormone-dependent women cancers. The microbiota can directly or indirectly modulate estrogen metabolism by altering systemic estrogen concentrations and the profile of its bioactive metabolites through mechanisms such as enterohepatic circulation and microbial enzymatic activity (e.g., β-glucuronidase). These alterations can, in turn, impact tumor development. Conversely, estrogen can shape the composition of the microbiota in gynecologic tumors, promoting the growth of *Lactobacillus* species and potentially affecting the tumor microenvironment and immune modulation. (11, 12).

**Figure 1:**
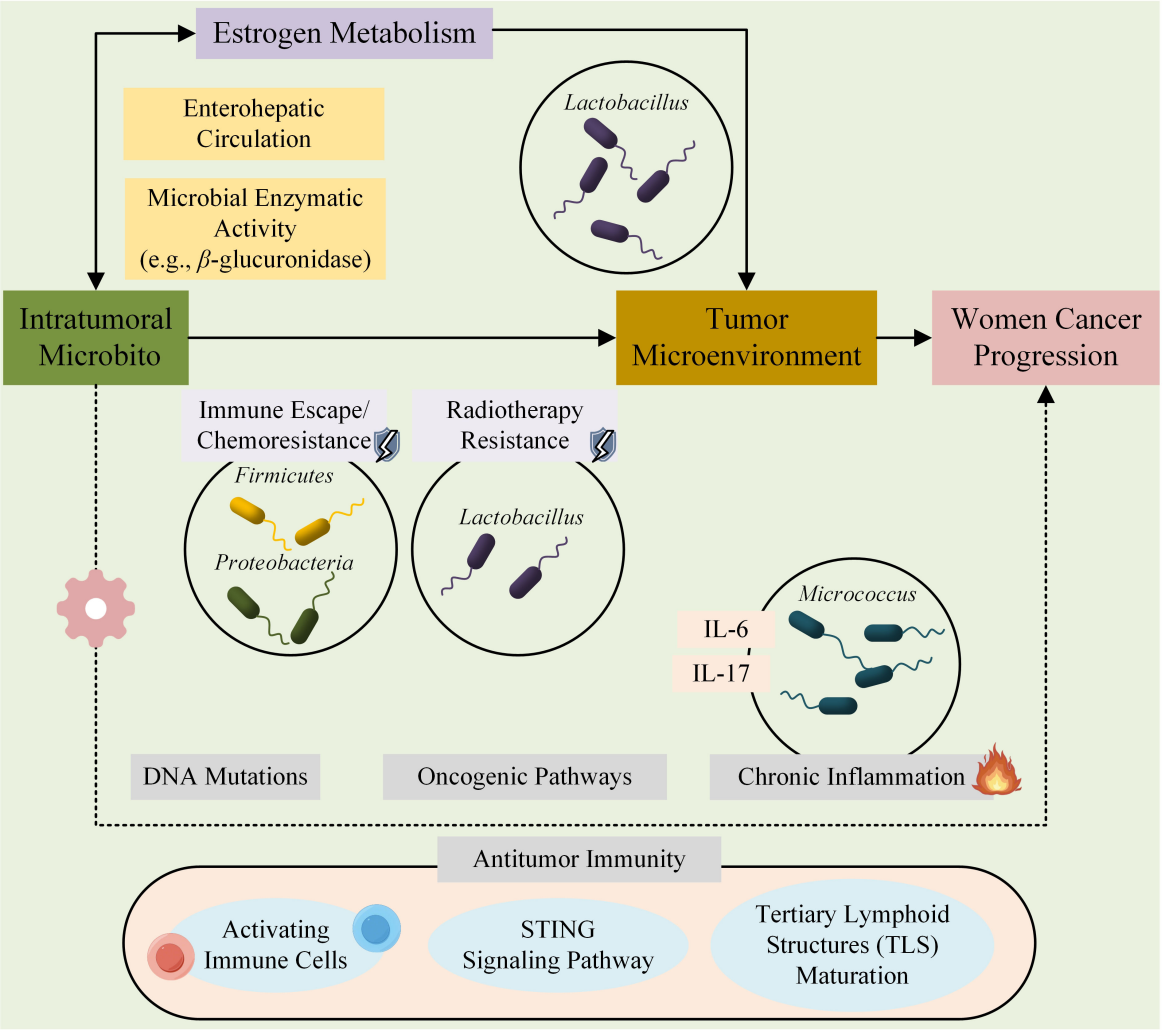
Proposed conceptual framework of the microbiota-estrogen-tumor microenvironment (TME) crosstalk in cancer progression. This schematic illustrates the complex and bidirectional interactions between the intratumoral microbiota, estrogen signaling, and components of the tumor microenvironment (TME). The microbiota can influence tumor development through multiple mechanisms, including modulation of estrogen metabolism via microbial enzymes (e.g., β-glucuronidase), induction of DNA damage, activation of oncogenic signaling pathways, and shaping of the immune landscape (e.g., through STING pathway activation, antigen presentation, and tertiary lymphoid structure formation). Conversely, estrogen may regulate the composition and function of the tumor-associated microbiota by promoting or inhibiting the growth of specific taxa (e.g., *Lactobacillus* spp.), thereby further influencing immune responses and tumor behavior. This tripartite interaction contributes to cancer initiation, progression, and treatment response in women’s cancers such as breast, ovarian, endometrial, and cervical cancers.

Despite increasing recognition of the interconnected roles of women cancers, intratumoral microbiota, and estrogen, systematic evaluations of the intratumoral microbiota across the four major types of women cancers—breast, ovarian, endometrial, and cervical—remain scarce. To address this gap, we conducted a systematic review to evaluate the composition of the intratumoral microbiota in women cancers. This review aims to explore the diversity, taxonomic abundance, and estrogen-mediated alterations in the intratumoral microbiota, as well as its potential role in tumor progression and therapeutic responses. To visually summarize the conceptual framework of this study, **Figure 1** illustrates the dynamic interplay between the intratumoral microbiota, estrogen, and the tumor microenvironment, highlighting our hypothesis that microbial–estrogen crosstalk plays a pivotal role in shaping tumor progression. Specifically, the diagram depicts (1) microbial modulation of estrogen metabolism and antitumor immunity, and (2) estrogen-driven feedback on microbial composition.

## 2 Methods

### 2.1 Search strategy

PubMed, Embase, Web of Science, and the Cochrane Library were searched from their inception to May 1, 2024, to identify the literature included in this systematic review. The search strategy is outlined in **Supplementary Table S1**. Search criteria were restricted to English-language publications, human studies, and those focused on women’s cancers. The systematic review was conducted in accordance with the PRISMA (Preferred Reporting Items for Systematic Reviews and Meta-analyses) guidelines (13), and the review protocol was pre-registered with PROSPERO (CRD 42024601213).

### 2.2 Selection of articles and data extraction

Studies included in this review adhered to the following inclusion criteria: 1) adult women (over 18 years old) who have undergone tissue sampling for gynecological cancer or breast cancer; 2) use of high-throughput sequencing to evaluate the diversity and composition of intratumoral microbiota; 3) analysis of microbial alterations across different disease states or treatment periods. Studies were excluded based on the following criteria: 1) articles related to *in vitro* studies, case reports, review articles, letters to the editor, comments, protocols, conference abstracts, or guidelines; 2) duplicate studies or overlapping study populations; 3) absence of a healthy or benign control group, or failure to evaluate microbiota composition.

Data from the selected studies were extracted as follows: study characteristics (first author’s name, publication year, study duration, country, participants, nationality, age, BMI, sample type, and estrogen receptor status or estrogen levels), sequence characteristics (sequencing technology, amplification region, and sequencing platform), and outcomes. This review primarily examines alterations in α-diversity, β-diversity, and relative abundance across various disease states, as well as the impact of antineoplastic therapy on the tumor microbiota.

### 2.3 Quality assessment

The Newcastle-Ottawa Scale (NOS) was employed to assess the quality of the included studies (14). The scale evaluates three key domains: selection, comparability, and exposure of cases and controls, yielding a maximum score of 9. Studies with scores below 6 were excluded from this systematic review. All included studies were independently assessed by two researchers, and any discrepancies were resolved through group consensus.

### 2.4 Data analysis

Estimated data from images were extracted using WebPlotDigitizer (15), and the estimated analysis of means (M) and standard deviations (SD) was calculated using an online calculator (http://www.math.hkbu.edu.hk/~tongt/papers/median2mean.html) (16). Data analysis was performed using Review Manager version 5.4. For continuous variables, standardized mean difference (SMD) and 95% confidence intervals (CI) were used to calculate effect sizes and represent variation in microbial diversity across studies. Heterogeneity was assessed using the I^2^ statistic, except in cases where only a single study was included, in which heterogeneity was not evaluated. An I^2^ value greater than 75% was considered to indicate substantial heterogeneity. In the presence of moderate to high heterogeneity (I² > 50%), a random-effects model was employed to obtain a more conservative and generalizable pooled estimate. When heterogeneity was low (I²< 50%), a fixed-effects model was considered. Given the overall heterogeneity observed among studies, a random-effects model was consistently applied throughout the analysis. Subgroup analyses stratified by tumor type were performed to explore potential sources of heterogeneity. Publication bias was assessed using funnel plots.

## 3 Results

### 3.1 Characteristics of included studies

**Figure 2:**
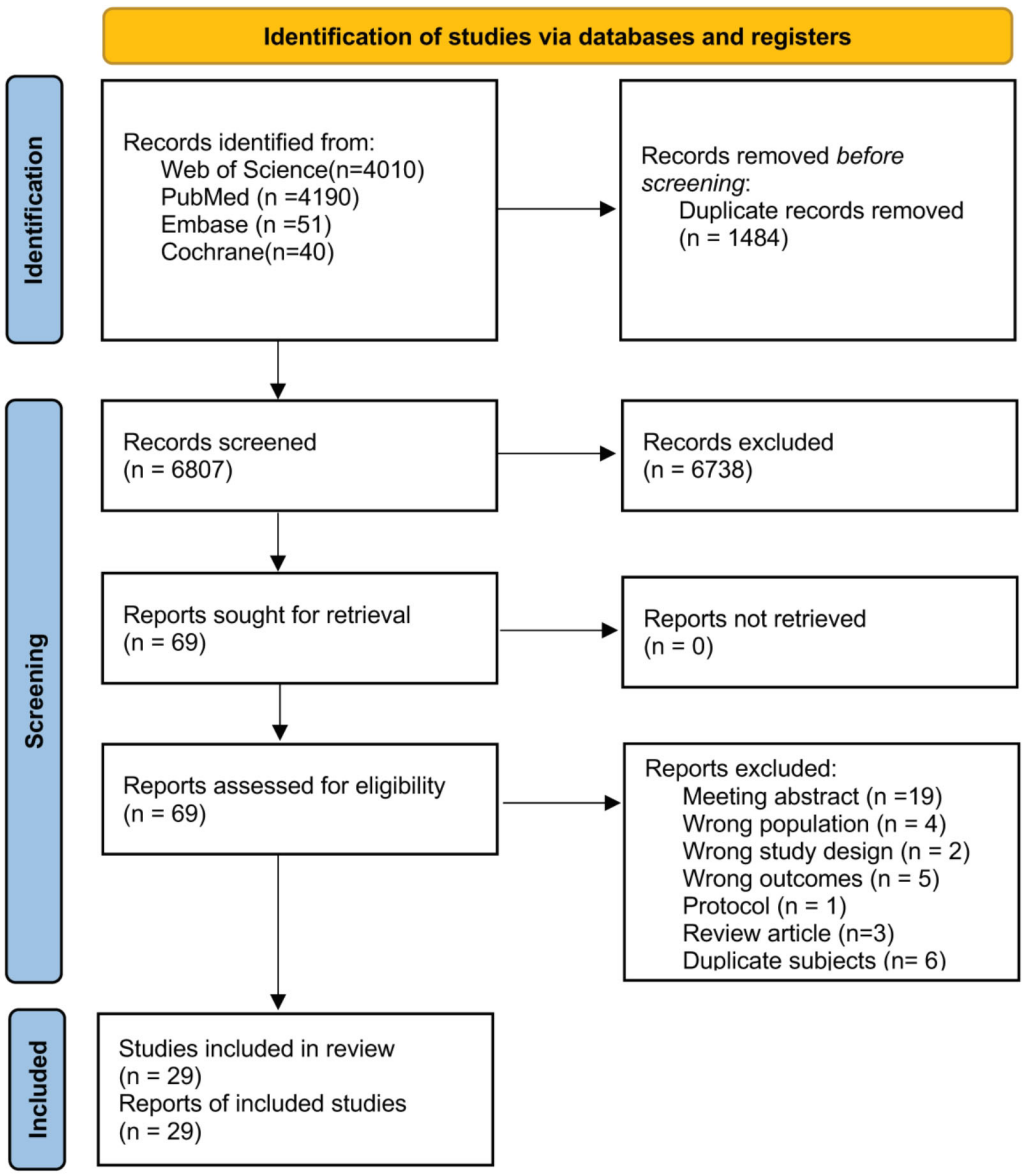
Flow diagram of the study selection process following the preferred reporting items for systematic reviews and meta-analyses (PRISMA) guidelines.

In total, 8,291 records were retrieved from the following databases: Web of Science (4,010), PubMed (4,190), Embase (51), and Cochrane Library (40). After a thorough examination of the titles, abstracts, and full texts, duplicates, irrelevant publications, and those containing inappropriate research content were excluded, resulting in the inclusion of 29 articles in the systematic review (**Figure 2**). Nineteen articles focused on breast cancer, three on ovarian cancer, six on endometrial cancer, and one on cervical cancer.

The characteristics of the included studies are summarized in **Table 1**. Thirteen studies were conducted in North America (12 in the USA and 1 in Canada), 11 in Asia (9 in China, 1 in Korea, and 1 in Israel), and 5 in Europe (3 in Italy, 1 in Ireland, and 1 in Slovakia). A total of 2,448 participants were enrolled in the research, comprising 99.41% females (n=2,007) and 0.59% males (n=12). In two studies, the sex of 429 participants was unspecified, and these participants were classified as female for subsequent analysis. The average age was 52.73 years, with a standard deviation of 12.36 years. The sample categories primarily included tumor tissue (n=1,322), normal tissue adjacent to the tumor mass (NAT, n=792), normal tissue (n=697), and benign disease tissue (n=157). NAT was defined as normal tissues located approximately 5 cm from the tumor. Regarding sequencing characteristics (**Supplementary Table S2**), 16S rRNA sequencing was the predominant method utilized (79.31%, n=23), followed by 16S rDNA (6.9%, n=2). The V4 region was the most frequently sequenced, appearing in 21 out of 29 studies, followed by V3 region in 20 out of 29 studies, and V1, V2, and V5 regions, each appearing in 7 out of 29 studies.

**Table 1:** Basic information included in the study.

Study	Country	Participants (n)	Mean age (y)	Sample type (n)	Estrogen (+)	Q*
Hogan-2021 (17)	Ireland	C: 23	70 (40-83)	TT: 23	21 (91.30%)	**7**
				match HT: 23		
				skin swab: 23		
Niccolai-2023 (18)	Italy	C: 20	F: 47 (IQR 41.66)	TT: 20	F: 9 (90%)	**8**
			M: 72 (IQR 55.82)	NAT: 20	M:10 (100%)	
Costantini-2018 (19)	Italy	C: 16	59 (46-82)	TT: 19	14 (87.5%)	**7**
				NAT: 19		
Thyagarajan-2020 (20)	USA	C: 23	27-78	TT: 23		**7**
				NAT: 23		
Hoskinson-2022 (21)	USA	C: 76	27-82	TT: 49		**7**
		H: 65		HT: 50		
				NAT: 51		
				pre-diagnose: 15		
Smith-2019 (22)	USA	C: 64	45 (18.72)	TT: 64		**7**
		H: 8		HT: 8		
				NAT: 11		
German-2023 (23)	USA	C: 76	C: 56	TT: 32	47 (62%)	**7**
		H: 403	H: 50	HT: 403		
				NAT: 61		
				MET: 9		
Tzeng-2021 (24)	USA	C: 221	C: 57 (47-66)	TT: 221	164 (82%)	**7**
		High risk: 18	High risk: 45(36-51)	High risk: 18		
		H: 69	H: 38 (26-47)	HT: 69		
				NAT: 221		
Nejman-2020 (25)	Isreal	C: 355	NA	TT: 355		**7**
		H: 54		HT: 54		
				NAT: 173		
Xuan-2014 (26)	USA	C: 20	63.8 ± 11.55	TT: 20	20 (100%)	**7**
				NAT: 20		
Esposito-2022 (27)	Italy	C: 34	>40: 11 (32.35%)	TT: 34		**8**
			≤40: 23 (67.65%)	NAT: 34		
Hieken-2016 (28)	USA	C: 15	B: 49 (33-70)	others: 33		**6**
		B:13	C: 75 (44-84)	NAT-tumor: 15		
				NAT-Benign: 13		
Klann-2020 (29)	USA	C: 10	NA	TT: 10		**6**
		H:10		HT: 36		
Urbaniak-2016 (30)	Canada	C: 45	C: 62 ± 16.40	TT: 35		**6**
		B: 13	H: 53 ± 12.05	HT: 23		
		H: 23		NAT: 13		
Kim-2021 (31)	Korean	C: 47	51.9 ± 10.7	TT: 47	28 (59.6%)	**7**
				NAT: 47		
				lymph node: 47		
Chiba-2020 (32)	USA	Pre-: 18	pre-: 65.3 ± 8.9	pre-: 18	pre-:39.0% post-: 21.5%	**6**
		Post-: 15	post-: 58.9 ± 10.1	post: 15		
Luo-2023 (33)	China	C: 18	C: 54 (43-90)	TT: 18	HR (**+**): 9(50%)	**7**
				NAT: 18		
		B: 8	B: 25 (18-35)	BT: 8		
Meng-2018 (34)	China	C: 72	C: 54 (29-77)	TT: 72	47	**6**
		B: 22	B: 47 (32-60)	BT: 22		
Hadzega-2021 (35)	Slovakia	C: 18	NA	TT: 18		**7**
		H: 5		HT: 5		
Zhou-2019 (36)	China	C: 25	C: 54.5 ± 7.3	TT: 25		**6**
		H: 25	H: 48.2 ± 7.7	HT*: 25		
Wang-2020 (37)	China	C: 6	C: 57.3 (46-75)	TT: 6		**6**
		H: 10	H: 51.6 (45-57)	non-cancer: 10		
Hawkins-2022 (38)	USA	C: 95	C: 64.47	TT: 95		**6**
		B: 16	B: 46	BT: 16		
Lu-2021 (8)	China	C: 25	C:≤50: 6 (24.00%)	TT: 25		**7**
			>50: 19 (76.00%)			
		B: 25	B:≤50: 19(76.0%)	BT: 25		
			>50: 6(24.00%)			
Wang-2022a (39)	China	C: 28	60.41 ± 5.22	TT: 28	Estrogen level: 23.51 ± 10.49 pg/ml	**8**
				NAT: 28		
Li-2021 (40)	China	C: 30	C: 56.4 ± 7.89	TT: 30		**7**
		H: 10	H: 53.1 ± 6.67	HT: 10		
				NAT: 30		
Walther-António-2016 (41)	USA	C: 17	C: 64 (58-71)	TT: 17		**6**
		B: 10	B: 44.5(42.5-52.5)	BT: 10		
		EH: 4	EH: 54(50.75-62.5)	EH: 4		
Walsh-2019 (42)	USA	C: 66	C: 61.8 (10.3)	TT: 16		**7**
		B: 75	B: 49.9 (10.5)	BT: 18		
		EH: 7	EH: 55.0 (3.3)			
Wang-2022b (43)	China	C: 10	C: 34 ± 5.68	TT: 10		**7**
		CIN1: 9	CIN1: 33 ± 4.23	CIN1: 9		
		CIN2: 11	CIN2: 33 ± 4.74	CIN2: 11		
		CIN3: 18	CIN3: 34 ± 5.68	CIN3: 18		
		H: 14	H: 32 ± 5.15	HT: 14		
Wang-2023 (44)	China	C: 10	C: 53.6 ± 9.67	TT: 10		**6**
		B: 10	B: 47.4 ± 8.75	BT: 10		

*Quality (Q) of each study was based on the Newcastle-Ottawa Quality. Bold values indicate the quality score assigned to each study. C, cancer; B, benign; H, health; F, female; M, male; pre-, pretreatment; post-, posttreatment; EH, endometrial hyperplasia; TT, tumor tissue; HT, healthy tissue; HT*, normal distal fallopian tube tissues; BT, benign tissue; NAT, normal tissue adjacent to the tumor mass; others, breast tissue; breast skin tissue; breast skin swabs; buccal swabs; pre-, before chemotherapy; post-, post-chemotherapy; HR, ER/PR.

### 3.2 α-diversity

#### 3.2.1 α-diversity alterations in different disease states

**Figure 3:**
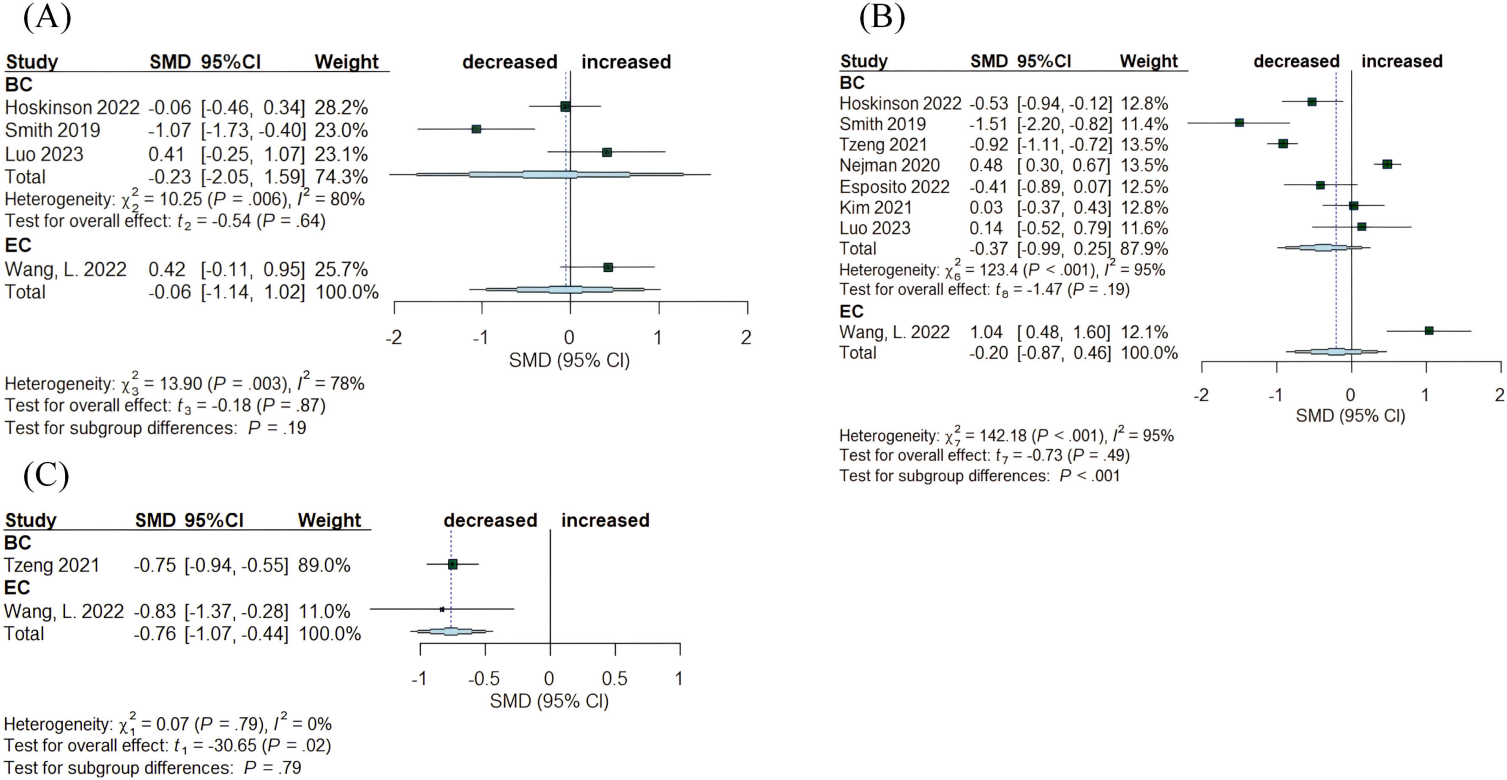
Forest plots illustrating alpha diversity indices comparing intratumoral microbiota with matched normal adjacent tissues (NATs) across female cancers. **(A)** Chao1 index; **(B)** Shannon index; **(C)** Simpson index. Each plot presents standardized mean differences (SMDs) with 95% confidence intervals (CIs), calculated using a random-effects model. Squares indicate individual study effect sizes (with sizes proportional to study weight); horizontal lines represent 95% CIs; diamonds denote pooled estimates. Positive SMD values indicate higher diversity in tumor tissues, while negative values indicate lower diversity compared to NATs. Statistical significance was defined as p < 0.05. BC, breast cancer; EC, endometrial cancer; CC, cervical cancer.

Twenty-two studies evaluated changes in α-diversity among women with cancer, disease-free controls, and individuals with benign disease. The sequencing reads originated mainly from tumor tissue, NAT, healthy control tissue (HC), and benign tissue. Species richness indexes, specifically the Chao1 index and observed species, along with metrics for richness and evenness such as the Shannon index and Simpson index, were the most commonly used measures to assess differences among disease states. The articles on breast cancer and endometrial cancer analyzed the distinctions between tumor tissue and adjacent normal tissue (**Figure 3**). In both cancer studies, species richness exhibited no significant alteration, with the Chao1 index values indicating SMD= -0.23, (95% CI: [-2.05,1.59], I^2^ = 80%) and SMD= 0.42, (95% CI: [-0.11, 0.95]), respectively (**Figure 3A**). The assessment of both richness and evenness using the Shannon index, evaluated in eight studies, revealed no significant changes (BC: SMD= -0.37, 95% CI: [-0.99, 0.25], I^2^ = 95%; EC: SMD= 1.04, 95% CI: [0.48, 1.60]) (**Figure 3B**). The Simpson index, although examined in only two studies, indicated a declining trend in both cancers (BC: SMD= -0.75, 95% CI: [-0.94, -0.55]; EC: SMD= -0.83, 95% CI: [-1.37, -0.28]) (**Figure 3C**).

**Figure 4:**
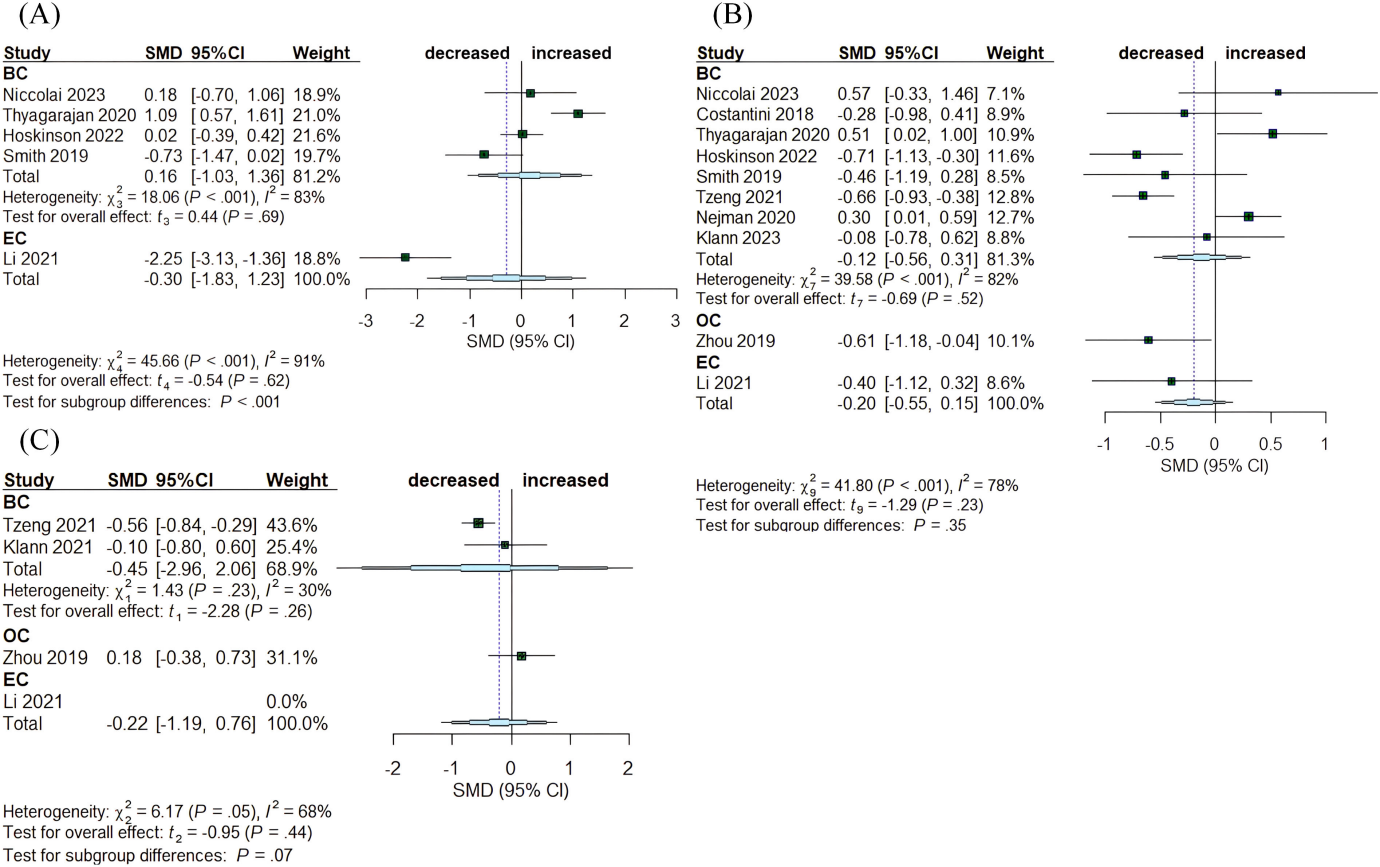
Forest plots illustrating alpha diversity indices comparing intratumoral microbiota with healthy controls (HC) across female cancers. **(A)** Chao1 index; **(B)** Shannon index; **(C)** Simpson index. Each plot presents standardized mean differences (SMDs) with 95% confidence intervals (CIs), calculated using a random-effects model. Squares indicate individual study effect sizes (with sizes proportional to study weight); horizontal lines represent 95% CIs; diamonds denote pooled estimates. Positive SMD values indicate higher diversity in tumor tissues, while negative values indicate lower diversity compared to HCs. Statistical significance was defined as p < 0.05. BC, breast cancer; EC, endometrial cancer; OC, ovarian cancer.

A total of ten articles compared tumor tissue with healthy normal tissue, encompassing breast, endometrial, and ovarian cancer (**Figure 4**). In BC, the Chao1 index exhibited no significant changes (SMD= 0.16, 95% CI: [-1.03,1.36], I^2^ = 83%), while in EC, the index demonstrated a reduction (SMD= -2.25, 95% CI: [-3.13, -1.36]) (**Figure 4A**). The Simpson and Shannon indices were computed for the three tumor types, revealing a decrease in the Shannon index for OC (SMD= -0.61, 95% CI: [-1.18, -0.04]). The other indices exhibited no significant changes: for BC, the Simpson index had an SMD of -0.45 (95% CI: [-2.96, 2.06], I^2^ = 30%) and the Shannon index had an SMD of -0.12, (95% CI: [-0.56, 0.31], I²= 82%); for OC, Simpson SMD= 0.18, (95% CI: [-0.38, 0.73]); for EC, Shannon SMD= -0.40, (95% CI: [-1.12, 0.32]) (**Figures 4C**, **B**).

**Figure 5:**
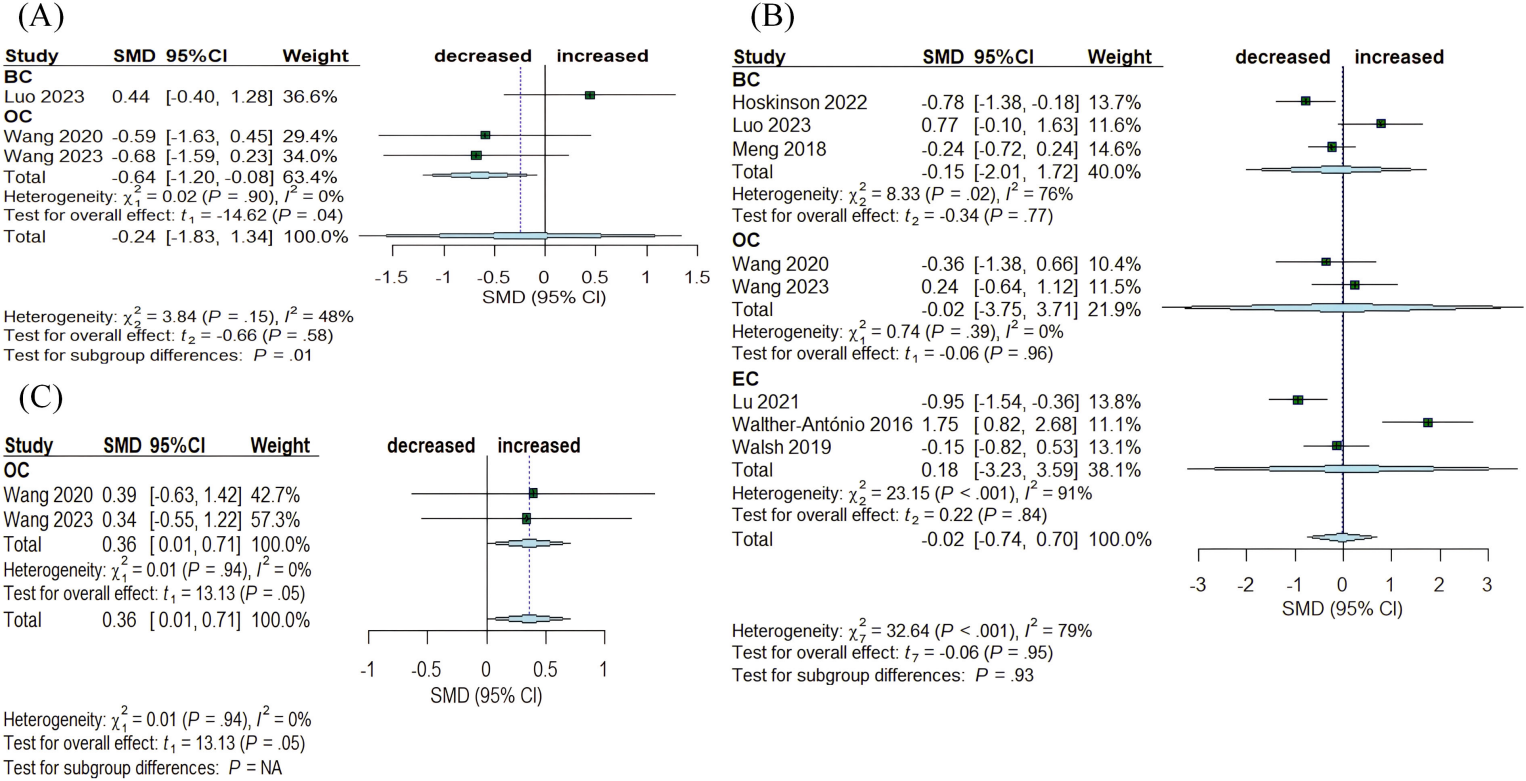
Forest plots illustrating alpha diversity indices comparing intratumoral microbiota with benign disease tissues across female cancers. **(A)** Chao1 index; **(B)** Shannon index; **(C)** Simpson index. Each plot presents standardized mean differences (SMDs) with 95% confidence intervals (CIs), calculated using a random-effects model. Squares indicate individual study effect sizes (with sizes proportional to study weight); horizontal lines represent 95% CIs; diamonds denote pooled estimates. Positive SMD values indicate higher diversity in tumor tissues, while negative values indicate lower diversity compared to benign disease tissues. Statistical significance was defined as p < 0.05. BC, breast cancer; EC, endometrial cancer; OC, ovarian cancer.

Benign diseases are frequently considered precancerous. Eight articles assessed the differences between benign and tumor tissue in the breast, ovary, and endometrium (**Figure 5**). The Chao1 index showed a decrease in OC (SMD= -0.64, 95% CI: [-1.20, -0.08], I^2^ = 0%), while no significant alterations were observed in BC (SMD= 0.44, 95% CI: [-0.40, 1.28]) (**Figure 5A**). Simpson index indicated an increasing trend in OC (SMD= 0.36, 95% CI: [0.01, 0.71], I^2^ = 0%) (**Figure 5C**). In contrast, Shannon index exhibited no significant changes across the three cancers: BC (SMD= -0.15, 95% CI: [-2.01, 1.72], I^2^ = 76%), OC (SMD= -0.02, 95% CI: [-3.75, 3.71], I^2^ = 0%), and EC (SMD= 0.18, 95% CI: [-3.23, 3.59], I^2^ = 91%) (**Figure 5B**).

#### 3.2.2 Analysis of publication bias

Given the limited sample size, the analysis for publication bias was restricted to the Shannon indices, employing funnel plots and Egger’s test. Funnel plots indicated an absence of significant publication bias (**Supplementary Figure S1**). Quantitative analysis utilizing Egger’s test indicated an absence of significant publication bias in comparisons of tumor tissue to normal tissue (t= 0.2146, p= 0.8345), normal tissue adjacent to tumor (t= -0.1101, p= 0.91), and benign tissue (t= 1.8306, p= 0.1169).

### 3.3 Microbial taxa abundance

**Figure 6:**
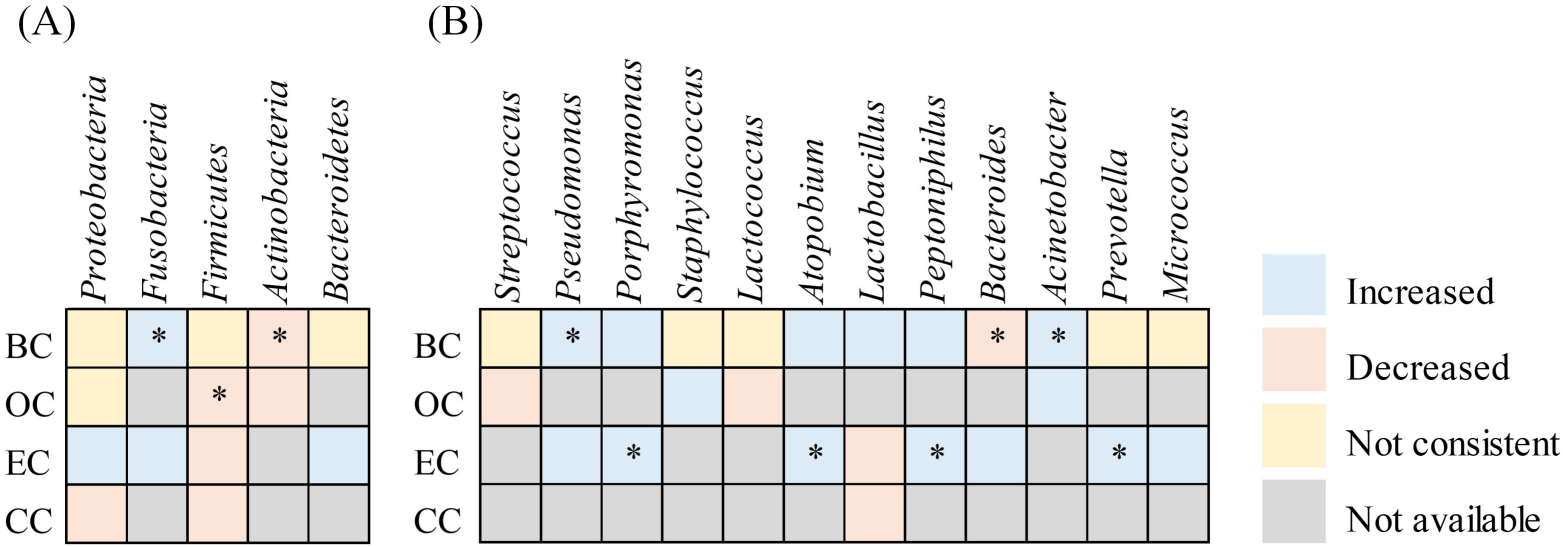
Heatmap summarizing changes in the relative abundance of microbial taxa in tumor tissues compared to non-tumor tissues across female cancer types. This heatmap illustrates reported increases or decreases in specific microbial taxa across endometrial cancer (EC), ovarian cancer (OC), breast cancer (BC), and cervical cancer (CC). An asterisk (*) denotes consistent findings reported in two or more independent studies.

Microbiota referenced in a minimum of three articles were incorporated into the analysis (**Figure 6**). *Proteobacteria*, *Firmicutes*, *Actinobacteria*, and *Bacteroidetes* represented the four predominant phyla across all tissues in BC, OC, EC, and CC. The microbiota abundance varied between tumor and non-tumor tissues. At the phylum level, four publications indicated that *Fusobacteriota* were enriched in tumor tissues, while five publications noted that *Firmicutes* and four publications highlighted that *Actinobacteria* were more abundant in non-tumor tissues. *Proteobacteria* and *Bacteroidetes* exhibited controversy, with four studies indicating a greater abundance of *Proteobacteria* in tumor tissues, while three reported the contrary. In contrast, two studies suggested a higher presence of *Bacteroidetes* in tumor tissue, with one presenting opposing evidence. At the genus level, *Pseudomonas* (n= 4), *Porphyromonas* (n= 3), *Atopobium* (n= 3), *Peptoniphilus* (n= 3) and *Acinetobacter* (n= 4) were significantly overrepresented in cancer tissues. Nonetheless, the prevalence of certain genera across various tissues continues to be a subject of debate, despite examination in multiple studies. *Streptococcus* shows 2 instances of increase compared to 3 instances of decrease; *Staphylococcus* exhibits 3 instances of increase versus 2 instances of decrease; *Lactococcus* has 1 instance of increase against 2 instances of decrease; *Lactobacillus* presents 1 instance of increase relative to 2 instances of decrease; *Bacteroides* indicates 1 instance of increase compared to 2 instances of decrease; *Prevotella* reflects 3 instances of increase alongside 3 instances of decrease; *Micrococcus* demonstrates 2 instances of increase in contrast to 1 instance of decrease.

### 3.4 Microbial alterations with estrogen

In BC, six studies assessed microbial changes associated with varying estrogen levels: three focused on estrogen receptor (ER) status, two on hormone receptor (HR) status (including ER or progesterone receptor (PR)), and one on menopause status. The genus *Alkanindiges* was negatively correlated with ER in two studies. Additionally, *Micrococcus*, *Caulobacter*, *Proteus*, *Brevibacillus*, *Kocuria*, *Parasediminibacterium*, *Comamonas*, and *Pseudoxanthomonas* were identified in one study. *Corynebacterium* was the sole genus positively associated with ER. Various HR statuses were also correlated with bacterial abundance. α-diversity and β-diversity did not exhibit significant differences across various HR statuses. In HR (-), *Acinetobacter*, *Priestia*, *Streptomyces*, *Rhodobacter*, *Bradyrhizobium*, *Pseudolysobacter*, *Gammaretrovirus*, *Adidovorax* exhibited higher abundance, while *Lawsonella*, *Spirosoma*, *Paracoccus*, *Actinomyces*, *Clostridium*, *Bacillus*, *Hydrogenophaga*, and *Staphylococcus*, along with *Halomonas*, were positively correlated with HR (+). A significantly increased relative abundance of *Acinetobacter* was noted in HR (+) breast tissue. Premenopause showed a higher abundance of *Ralstonia*, *Acetobacter aceti*, *Lactobacillus vini*, *Lactobacillus paracasei*, and *Xanthomonas* sp. in comparison to the postmenopausal period.

Correlation analysis for EC indicated that the genera *Dialister*, *Rhodococcus*, *Delftia*, and *Parvimonas* exhibited a positive correlation with estrogen levels (p< 0.05). Additionally, *A. tetradius*, *A. lactolyticus*, *P. coxii*, and *C. ureolyticus* were found to be associated with EC and postmenopause.

### 3.5 Microbial alterations with treatment

Only one study examined the effect of antineoplastic therapy on intratumoral microbiota (32). Chiba et al. reported that the neoadjuvant chemotherapy significantly decreased the diversity of intratumor microbiota by comparing breast cancer patients who underwent neoadjuvant chemotherapy (n= 15) with those who did not (n= 18) (Shannon index: SMD= -0.95, 95% CI: [- 1.68, -0.22]). Additionally, neoadjuvant chemotherapy-induced alterations in the composition of intratumoral microbiota, were notably characterized by a significant increase in the abundance of the genus *Pseudomonas* and a decrease in the genus *Prevotella* (P< 0.05).

## 4 Discussion

Previous studies have identified distinct microbiota profiles in various tumor tissues, which are associated with clinical characteristics such as tumor stage and estrogen levels (25). This systematic review aims to evaluate prevalent changes in the intratumoral microbiota associated with estrogen in female cancers. Our findings revealed alterations in the α-diversity and composition of the intratumoral microbiota, which were linked to estrogen and antitumor therapy.

α-diversity was assessed primarily using the Chao1, Shannon, and Simpson indices. Variations in α-diversity have not been consistently reported in previous studies, with literature indicating increases, decreases, and instances of no significant changes in diversity. Our study indicated that the Simpson index was the only measure exhibiting a decreasing trend in both BC (SMD= -0.75, 95% CI: [-0.94, -0.55]) and EC (SMD= -0.83, 95% CI: [-1.37, -0.28]) when comparing tumor tissues to adjacent normal tissues. In comparisons of tumor tissues with normal tissues, the Chao1 index showed a reduction in EC (SMD= -2.25, 95% CI: [-3.13, -1.36]) while the Shannon index revealed a decrease in OC (SMD= -0.61, 95% CI: [-1.18, -0.04]). When comparing tumor tissues to benign tissues, the Chao1 index revealed a decrease in OC (SMD= -0.64, 95% CI: [-1.20, -0.08], I^2^ = 0%), while the Simpson index demonstrated an increase in OC (SMD= 0.36, 95% CI: [0.01, 0.71], I^2^ = 0%). Other indices exhibited no significant differences between tumor and non-tumor tissues. The reduction in α-diversity in tumor tissues compared to non-tumor tissues suggests that dysbiosis may play a role in tumor development. Given the limited number of included studies and the potential influence of various factors on the intratumoral microbiota, a larger sample size may be required to confirm changes in α-diversity. Moreover, despite performing subgroup analyses based on tumor type, significant heterogeneity remained, which may be attributed to the inclusion of study populations with varying stages, grades, races, and hormonal statuses.

Additionally, the composition of intratumoral microbiota displayed statistically significant variations across different tissue types. At the phylum level, *Fusobacteriota* were consistently reported to be enriched in tumor tissues across four studies, whose critical role were previously highlighted in the tumor microenvironment of various cancer types, particularly colorectal cancer. The presence of *Fusobacterium* in intratumor colonization influences the immune response, tumor cell proliferation, and drug resistance. Although research on *Fusobacterium* in women’s cancers is limited, a negative correlation has been observed between the abundance of *Fusobacterium nucleatum* in cervical cancer tissues and prognosis (45). Furthermore, an animal model of breast cancer demonstrated that *Fusobacterium nucleatum* binds to breast cancer tissues via its leptin Fap2, inhibiting the accumulation of tumor-infiltrating T cells and thereby promoting tumor growth and metastatic progression (46).

At the genus level, *Pseudomonas*, *Porphyromonas*, *Atopobium*, *Peptoniphilus*, and *Acinetobacter* were consistently found to be enriched in tumor tissues across multiple studies. These microbial signatures may hold functional relevance for tumor behavior and therapeutic response. For example, *Pseudomonas aeruginosa* has demonstrated anti-proliferative properties, with its ExoT effector protein shown to impede tumor cell division. A randomized, double-blind, placebo-controlled trial is currently assessing *Pseudomonas aeruginosa*-mannose sensitive hemagglutinin (PA-MSHA) as a neoadjuvant agent in HER2-negative breast cancer, with preliminary findings suggesting clinical benefit (47). These observations raise the possibility that the intratumoral enrichment of *Pseudomonas* may serve as a favorable prognostic indicator in specific contexts. In contrast, other taxa appear to promote oncogenic processes. *Porphyromonas gingivalis*, for instance, can enhance tumor invasiveness by stimulating IL-8 secretion and promoting IL-8-dependent matrix metalloproteinase (MMP) activity (48). Furthermore, *Atopobium vaginae* and *Porphyromonas somerae* have been shown to induce proinflammatory cytokines—such as IL-1α, IL-1β, IL-17α, and TNFα—when co-cultured with endometrial cells, potentially contributing to a pro-tumorigenic inflammatory microenvironment (49). Collectively, these findings indicate that bacterial enrichment in tumor tissues may influence carcinogenesis through distinct molecular pathways, including immune modulation, cytokine-driven inflammation, and matrix remodeling. These microbe-host interactions may also intersect with hormonal signaling and therapeutic exposures, particularly in hormone-sensitive malignancies such as breast and endometrial cancers. Future studies should aim to delineate these pathway-specific effects and evaluate their potential utility as biomarkers or therapeutic targets.

In addition to microbial composition and diversity, recent studies have highlighted the critical role of microbial metabolic functions and immune modulation in tumor biology. Of particular interest is the estrobolome - a collection of microbial genes involved in estrogen metabolism - which has been implicated in the regulation of systemic estrogen levels and the pathogenesis of hormone-driven malignancies such as breast and endometrial cancer (50). Dysbiosis within the estrobolome may disrupt estrogen homeostasis, potentially contributing to oncogenesis or resistance to therapy. Estrogen itself, a pivotal hormone in female reproductive physiology, is known to drive the development of hormone-sensitive cancers when present at dysregulated or excessive levels. Increasing evidence suggests that estrogen homeostasis is partially governed by the gut microbiota through mechanisms such as deconjugation and enterohepatic recirculation, thereby influencing systemic estrogen exposure (51, 52). However, the relationship between intratumoral microbiota and estrogen signaling remains underexplored. Limited but notable studies have reported tumor-specific microbial shifts associated with estrogenic states. In BC, the genera *Alkanindiges*, *Micrococcus*, *Caulobacter*, *Proteus*, *Brevibacillus*, *Kocuria*, *Parasediminibacterium*, *Comamonas*, and *Pseudoxanthomonas* exhibited a decrease in ER (+) tumors, while *Corynebacterium* showed increased abundance. In EC, genera such as *Dialister*, *Rhodococcus*, *Delftia*, and *Parvimonas* were enriched in patients with elevated estrogen levels, whereas *A. tetradius*, *A. lactolyticus*, *P. coxii*, and *C. ureolyticus* were more prevalent in postmenopausal women. These findings hint at a context-specific microbial modulation potentially linked to estrogen availability, yet inconsistencies across studies limit the ability to draw definitive conclusions. Further research is warranted to delineate the functional pathways through which intratumoral microbiota may influence tumor behavior in estrogen-dependent cancers. Potential mechanisms may include microbial regulation of local estrogen metabolism, modulation of hormone receptor expression, or interactions with estrogen-responsive immune pathways.

While numerous studies have investigated the role of microbiota in antitumor efficacy (53), relatively few have explored the bidirectional interactions between intratumor microbiota and antitumor therapy. Intratumoral microbiota may influence the effectiveness of antineoplastic treatment by modulating antitumor immunity through shaping the tumor microenvironment. Previous murine models have demonstrated that the antitumor efficacy of gemcitabine was diminished in *Mycoplasma hyorhinis*-infected murine mammary tumors compared to uninfected murine mammary tumors (54). Conversely, antitumor therapy can reshape the tumor microenvironment, thereby affecting the composition and diversity of the intratumoral microbiota. Chiba et al. observed that breast cancer patients undergoing neoadjuvant chemotherapy exhibited decreased microbial diversity within tumors compared to untreated patients. Moreover, emerging evidence suggests that microbiota can influence immune checkpoint activity, including PD-1/PD-L1 signaling, potentially shaping responsiveness to immunotherapy (55). These therapy-induced microbial changes may be associated with adverse treatment effects or tumor recurrence (56, 57). Notably, such interactions may be subtype-specific, as microbial compositions have been shown to differ between luminal and triple-negative breast cancer, and between endometrioid and serous subtypes of endometrial cancer (9).

Several limitations exist in this study. First, the small sample sizes in the included studies undermine the robustness and generalizability of the findings. Few studies have investigated the effects of estrogen levels and antitumor therapy on intratumoral microbiota, highlighting the need for further research with larger sample sizes. Second, the inclusion of only English-language literature may introduce publication bias, which may have limited the comprehensiveness of the findings. Third, there was substantial heterogeneity observed in the diversity analyses, which may be attributable to clinical factors such as ethnicity, cancer stage, and treatment regimen. However, due to the lack of consistently reported data on these variables across the included studies, subgroup meta-analyses could not be performed to explore their potential contributions to the heterogeneity. An additional important limitation is that several included studies did not clearly report the sex of their participants. In these cases, the sex of participants was inferred as female based on contextual cues, which may have introduced classification bias. This limitation may reduce the generalizability of the findings and underscores the need for future studies to provide explicit sex-disaggregated data.

Overall, our study revealed notable alterations in the composition of the intratumoral microbiota in women’s cancers, particularly in relation to estrogenic status and exposure to antitumor therapies. These findings suggest that hormonal regulation and therapeutic interventions may reshape the tumor-associated microbial landscape, potentially influencing tumor progression and treatment response. Further investigations are warranted to elucidate the microbial metabolic pathways, host-microbe interactions, and immune modulatory roles involved, which could help identify novel biomarkers or therapeutic targets for precision oncology.

## 5 Conclusion

In summary, this systematic review highlights distinct alterations in the intratumoral microbiota associated with female malignancies. Notably, a consistent enrichment of *Fusobacteriota* was observed in tumor tissues, while *Firmicutes* and *Actinobacteria* were more abundant in adjacent non-tumor tissues. Reductions in α-diversity were frequently reported in tumor samples, suggesting a less diverse microbial community within the tumor microenvironment. In addition, estrogen-related microbial shifts - such as the increased prevalence of *Dialister*, *Rhodococcus*, and *Parvimonas* under elevated estrogenic states - and treatment-induced changes, including reduced microbial diversity following chemotherapy, were observed. These findings point to a potential role of the intratumoral microbiota in modulating tumor progression and treatment response. Future mechanistic studies are warranted to elucidate the functional contributions of specific microbial taxa, which may ultimately aid in the development of targeted microbiome-based strategies for cancer therapy and women’s health.

## Data Availability

The original contributions presented in the study are included in the article/**Supplementary Material**. Further inquiries can be directed to the corresponding authors.
