# Supplementary material for: Intratumoral microbiota composition in women’s cancers: a systematic review and meta-analysis
Source: Front Oncol. 2025 Jun 12;15:1544786. doi: 10.3389/fonc.2025.1544786 (PMC12197917; doi:10.3389/fonc.2025.1544786)
Supplement: Supplementary file 1 [file DataSheet1.docx]

Supplementary Material

# Supplementary Tables

**Table S1 Electronic search strategy in PubMed, Embase, Web of Science and Cochrane Library databases**

**1.1 PubMed**

| ID | Search | results |
| --- | --- | --- |
| #1  cervical tissue | ((cervical) OR (cervix)) AND ((tissue) OR (biopsy) OR (sample)) |  |
| #2  endometrial tissue | ((endometrial) OR (endometrium)) AND ((tissue) OR (biopsy) OR (sample)) |  |
| #3  ovarian tissue | ((ovarian) OR (ovary)) AND ((tissue) OR (biopsy) OR (sample)) |  |
| #4  breast tissue | ((breast) OR (mammary)) AND ((tissue) OR (biopsy) OR (sample)) |  |
| #5  microbiome | ((microbiome) OR (microbiota) OR (ecosystem) OR (bacteria) OR (flora) OR (microflora) OR (dysbiosis)) |  |
| #6  sequence | ((16S) OR (Sequencing) OR (Shotgun) OR (Metagenome) OR (NGS) OR (illumina) OR (pyrosequencing)) |  |
| #7  cervical cancer | ((cervical) OR (cervix)) AND ((cancer) OR (carcinoma) OR (neoplasm) OR (tumor) OR (malignancy)) |  |
| #8  endometrial cancer | ((endometrial) OR (endometrium)) AND ((cancer) OR (carcinoma) OR (neoplasm) OR (tumor) OR (malignancy)) |  |
| #9  ovarian cancer | ((ovarian) OR (ovary)) AND ((cancer) OR (carcinoma) OR (neoplasm) OR (tumor) OR (malignancy)) |  |
| #10  breast cancer | ((breast) OR (mammary)) AND ((cancer) OR (carcinoma) OR (neoplasm) OR (tumor) OR (malignancy)) |  |
| #11 | ((humans [Filter]) AND (1000/1/1:2024/5/1[pdat]) AND (English [Filter])) |  |
| #12 | #1 AND #5 AND #6 AND #7 AND #11 | 2801 |
| #13 | #2 AND #5 AND #6 AND #8 AND #11 | 103 |
| #14 | #3 AND #5 AND #6 AND #9 AND #11 | 281 |
| #15 | #4 AND #5 AND #6 AND #10 AND #11 | 1005 |

**1.2 Embase**

| ID | Search | results |
| --- | --- | --- |
| #1  cervical tissue | 'cervical tissue'/exp OR 'cervical tissue':ti,ab,kw OR 'cervical biopsy':ti,ab,kw OR 'cervical sample':ti,ab,kw OR 'cervix tissue':ti,ab,kw OR 'cervix biopsy':ti,ab,kw OR 'cervix sample':ti,ab,kw |  |
| #2  endometrial tissue | 'endometrial tissue'/exp OR 'endometrial tissue':ti,ab,kw OR 'endometrial biopsy':ti,ab,kw OR 'endometrial sample':ti,ab,kw OR 'endometrium tissue':ti,ab,kw OR 'endometrium biopsy':ti,ab,kw OR 'endometrium sample':ti,ab,kw |  |
| #3  ovarian tissue | 'ovarian tissue'/exp OR 'ovarian tissue':ti,ab,kw OR 'ovarian biopsy':ti,ab,kw OR 'ovarian sample':ti,ab,kw OR 'ovary tissue':ti,ab,kw OR 'ovary biopsy':ti,ab,kw OR 'ovary sample':ti,ab,kw |  |
| #4  breast tissue | 'breast tissue'/exp OR 'breast tissue':ti,ab,kw OR ' breast biopsy':ti,ab,kw OR ' breast sample':ti,ab,kw OR 'mammary tissue':ti,ab,kw OR ' mammary biopsy':ti,ab,kw OR ' mammary sample':ti,ab,kw |  |
| #5  Microbiome | 'microbiome':ti,ab,kw OR 'microbiota':ti,ab,kw OR 'ecosystem':ti,ab,kw OR 'bacteria':ti,ab,kw OR 'flora':ti,ab,kw OR 'microflora':ti,ab,kw OR 'dysbiosis':ti,ab,kw |  |
| #6  Sequence | '16s':ti,ab,kw OR 'sequencing':ti,ab,kw OR 'shotgun':ti,ab,kw OR 'metagenome':ti,ab,kw OR ' NGS ':ti,ab,kw OR 'illumina':ti,ab,kw OR 'pyrosequencing':ti,ab,kw |  |
| #7  Cervical cancer | 'uterine cervix carcinoma'/exp OR 'uterine cervix cancer'/exp OR 'cervical cancer':ti,ab,kw OR 'cervical carcinoma':ti,ab,kw OR 'cervical neoplasm':ti,ab,kw OR 'cervical tumor':ti,ab,kw OR 'cervical malignancy':ti,ab,kw OR 'cervix cancer':ti,ab,kw OR 'cervix carcinoma':ti,ab,kw OR 'cervix neoplasm':ti,ab,kw OR 'cervix tumor ':ti,ab,kw OR 'cervix malignancy':ti,ab,kw |  |
| #8  endometrial cancer | 'endometrial neoplasms'/exp OR 'endometrial cancer'/exp OR 'endometrial cancer':ti,ab,kw OR 'endometrial carcinoma':ti,ab,kw OR 'endometrial neoplasm':ti,ab,kw OR 'endometrial tumor':ti,ab,kw OR 'endometrial malignancy':ti,ab,kw OR 'endometrium cancer':ti,ab,kw OR 'endometrium carcinoma':ti,ab,kw OR 'endometrium neoplasm':ti,ab,kw OR 'endometrium tumor ':ti,ab,kw OR 'endometrium malignancy':ti,ab,kw |  |
| #9  ovarian cancer | 'ovarian neoplasms'/exp OR 'ovarian cancer'/exp OR 'ovarian cancer':ti,ab,kw OR 'ovarian carcinoma':ti,ab,kw OR 'ovarian neoplasm':ti,ab,kw OR 'ovarian tumor':ti,ab,kw OR 'ovarian malignancy':ti,ab,kw OR 'ovary cancer':ti,ab,kw OR 'ovary carcinoma':ti,ab,kw OR 'ovary neoplasm':ti,ab,kw OR 'ovary tumor ':ti,ab,kw OR 'ovary malignancy':ti,ab,kw |  |
| #10  breast cancer | 'breast neoplasms'/exp OR 'breast cancer'/exp OR 'breast cancer':ti,ab,kw OR 'breast carcinoma':ti,ab,kw OR 'breast neoplasm':ti,ab,kw OR 'breast tumor':ti,ab,kw OR 'breast malignancy':ti,ab,kw OR 'mammary cancer':ti,ab,kw OR 'mammary carcinoma':ti,ab,kw OR 'mammary neoplasm':ti,ab,kw OR 'mammary tumor ':ti,ab,kw OR 'mammary malignancy':ti,ab,kw |  |
| #11 | #1 AND #5 AND #6 AND #7 AND [english]/lim AND [humans]/lim AND [embase]/lim AND [01-01-1000]/sd NOT [05-01-2024]/sd | 0 |
| #12 | #2 AND #5 AND #6 AND #8 AND [english]/lim AND [humans]/lim AND [embase]/lim AND [01-01-1000]/sd NOT [05-01-2024]/sd | 5 |
| #13 | #3 AND #5 AND #6 AND #9 AND [english]/lim AND [humans]/lim AND [embase]/lim AND [01-01-1000]/sd NOT [05-01-2024]/sd | 5 |
| #14 | #4 AND #5 AND #6 AND #10 AND [english]/lim AND [humans]/lim AND [embase]/lim AND [01-01-1000]/sd NOT [05-01-2024]/sd | 41 |

**1.3 Web of Science**

| ID | Search | results |
| --- | --- | --- |
| #1  cervical tissue | ((TS=(cervical tissue)) OR (TS=(cervical biopsy)) OR (TS=(cervical sample)) OR (TS=(cervix tissue)) OR (TS=(cervix biopsy)) OR (TS=(cervix sample))) |  |
| #2  endometrial tissue | ((TS=(endometrial tissue)) OR (TS=(endometrial biopsy)) OR (TS=(endometrial sample)) OR (TS=(endometrium tissue)) OR (TS=(endometrium biopsy)) OR (TS=(endometrium sample))) |  |
| #3  ovarian tissue | ((TS=(ovarian tissue)) OR (TS=(ovarian biopsy)) OR (TS=(ovarian sample)) OR (TS=(ovary tissue)) OR (TS=(ovary biopsy)) OR (TS=(ovary sample))) |  |
| #4  breast tissue | ((TS=(breast tissue)) OR (TS=(breast biopsy)) OR (TS=(breast sample)) OR (TS=(mammary tissue)) OR (TS=(mammary biopsy)) OR (TS=(mammary sample))) |  |
| #5  Microbiome | ((TS=(microbiome)) OR (TS=(microbiota)) OR (TS=(ecosystem)) OR (TS=(bacteria)) OR (TS=(flora)) OR (TS=(microflora)) OR (TS=(dysbiosis))) |  |
| #6  Sequence | ((TS=(16s)) OR (TS=(sequencing)) OR (TS=(shotgun)) OR (TS=(metagenome)) OR (TS=(NGS)) OR (TS=(illumina)) OR (TS=(pyrosequencing))) |  |
| #7  Cervical cancer | ((TS=(cervical cancer)) OR (TS=(cervical carcinoma)) OR (TS=(cervical neoplasm*)) OR (TS=(cervical tumor)) OR (TS=(cervical malignancy)) OR (TS=(cervix cancer)) OR (TS=(cervix carcinoma)) OR (TS=(cervix neoplasm*)) OR (TS=(cervix tumor)) OR (TS=(cervix malignancy)) OR (TS=(Uterine Cervical Neoplasm*)) OR (TS=(Cancer of the Uterine Cervix)) OR (TS=(Cancer of the Cervix)) OR (TS=(Uterine Cervical Cancer*)) OR (TS=(Cancer of Cervix))) |  |
| #8  endometrial cancer | ((TS=(endometrial cancer)) OR (TS=(endometrial carcinoma)) OR (TS=(endometrial neoplasm*)) OR (TS=(endometrial tumor)) OR (TS=(endometrial malignancy)) OR (TS=(endometrium cancer)) OR (TS=(endometrium carcinoma)) OR (TS=(endometrium neoplasm*)) OR (TS=(endometrium tumor)) OR (TS=(endometrium malignancy))) |  |
| #9  ovarian cancer | ((TS= (ovarian cancer)) OR (TS=(ovarian carcinoma)) OR (TS=(ovarian neoplasm*)) OR (TS=(ovarian tumor)) OR (TS=(ovarian malignancy)) OR (TS=(ovary cancer)) OR (TS= (ovary carcinoma)) OR (TS=(ovary neoplasm*)) OR (TS=(ovary tumor)) OR (TS= (ovary malignancy))) |  |
| #10  breast cancer | ((TS= (breast cancer)) OR (TS=(breast carcinoma)) OR (TS=(breast neoplasm*)) OR (TS=(breast tumor)) OR (TS=(breast malignancy)) OR (TS=(mammary cancer)) OR (TS=(mammary carcinoma)) OR (TS=(mammary neoplasm*)) OR (TS=(mammary tumor)) OR (TS=(mammary malignancy))) |  |
| #11 | #1 AND #5 AND #6 AND #7 AND DOP= (1972-01-01/2024-05-01) and Humans (MeSH Headings) and English (Languages) | 2385 |
| #12 | #2 AND #5 AND #6 AND #8 AND DOP= (1972-01-01/2024-05-01) and Humans (MeSH Headings) and English (Languages) | 85 |
| #13 | #3 AND #5 AND #6 AND #9 AND DOP= (1972-01-01/2024-05-01) and Humans (MeSH Headings) and English (Languages) | 367 |
| #14 | #4 AND #5 AND #6 AND #10 AND DOP= (1972-01-01/2024-05-01) and Humans (MeSH Headings) and English (Languages) | 1173 |

**1.4 Cochrane library**

| ID | Search | results |
| --- | --- | --- |
| #1  cervical tissue | (cervical tissue) OR (cervical biopsy) OR (cervical sample) OR (cervix tissue) OR (cervix biopsy) OR (cervix sample) |  |
| #2  endometrial tissue | (endometrial tissue) OR (endometrial biopsy) OR (endometrial sample) OR (endometrium tissue) OR (endometrium biopsy) OR (endometrium sample) |  |
| #3  ovarian tissue | (ovarian tissue) OR (ovarian biopsy) OR (ovarian sample) OR (ovary tissue) OR (ovary biopsy) OR (ovary sample) |  |
| #4  breast tissue | (breast tissue) OR (breast biopsy) OR (breast sample) OR (mammary tissue) OR (mammary biopsy) OR (mammary sample) |  |
| #5  Microbiome | (microbiome) OR (microbiota) OR (ecosystem) OR (bacteria) OR (flora) OR (microflora) OR (dysbiosis) |  |
| #6  sequence | (16s) OR (sequencing) OR (shotgun) OR (metagenome) OR (NGS) OR (illumine) OR (pyrosequencing) |  |
| #7  Cervical cancer | (cervical cancer) OR (cervical carcinoma) OR (cervical neoplasm) OR (cervical tumor) OR (cervical malignancy) OR (cervix cancer) OR (cervix carcinoma) OR (cervix neoplasm) OR (cervix tumor) OR (cervix malignancy) |  |
| #8  endometrial cancer | (endometrial cancer) OR (endometrial carcinoma) OR (endometrial neoplasm) OR (endometrial tumor) OR (endometrial malignancy) OR (endometrium cancer) OR (endometrium carcinoma) OR (endometrium neoplasm) OR (endometrium tumor) OR (endometrium malignancy) |  |
| #9  ovarian cancer | (ovarian cancer) OR (ovarian carcinoma) OR (ovarian neoplasm) OR (ovarian tumor) OR (ovarian malignancy) OR (ovary cancer) OR (ovary carcinoma) OR (ovary neoplasm) OR (ovary tumor) OR (ovary malignancy) |  |
| #10  breast cancer | (breast cancer) OR (breast carcinoma) OR (breast neoplasm) OR (breast tumor) OR (breast malignancy) OR (mammary cancer) OR (mammary carcinoma) OR (mammary neoplasm) OR (mammary tumor) OR (mammary malignancy) |  |
| #11 | Filter: date:1949/01/01-2024/05/01 |  |
| #12 | Filter: language: English |  |
| #13 | #1 AND #5 AND #6 AND #7 AND #11 AND #12 in Cochrane Trials | 11 |
| #14 | #2 AND #5 AND #6 AND #8 AND #11 AND #12 in Cochrane Trials | 1 |
| #15 | #3 AND #5 AND #6 AND #9 AND #11 AND #12 in Cochrane Trials | 4 |
| #16 | #4 AND #5 AND #6 AND #10 AND #11 AND #12 in Cochrane Trials | 24 |

**Table S2**. Detailed characteristics of the included studies

| **Study** | **Cancer type** | **Technology employed** | **amplified region** | **Platform** |
| --- | --- | --- | --- | --- |
| Hogan-2021[1] | BC | 16S rRNA | V3-V4 | Illumina MiSeq |
| Niccolai-2023[2] | BC | 16S rRNA | V3-V4 | Illumina MiSeq |
| Costantini-2018[3] | BC | 16S rRNA | V3 | Ion PGM Sequencer |
| Thyagarajan-2020[4] | BC | 16S rRNA | V3-V4 | Illumina MiSeq |
| Hoskinson-2022[5] | BC | 16S rRNA | V3-V4 | Illumina MiSeq |
| Smith-2019[6] | BC | 16S rRNA | V4 | Illumina MiSeq |
| German-2023[7] | BC | 16S rRNA | V1V2, V2V3, V3V4, | Illumina MiSeq |
|  |  |  | V4V5, V5V7, V7V9 |  |
| Tzeng-2021[8] | BC | 16S rRNA | V3-V4, V7-V9 | Illumina MiSeq |
| Nejman-2020[9] | BC | 16S rRNA | V4 or V3-V4 | Illumina Hi-seq 2500 |
| Xuan-2014[10] | BC | 16S pyrosequencing | 16S V4 rDNA | Illumina Miseq |
| Esposito-2022[11] | BC | 16S rRNA | V4-V6 | Illumina MiSeq |
| Hieken-2016[12] | BC | 16S rRNA | V3-V5 | Illumina MiSeq |
| Klann-2020[13] | BC | 16S rRNA | V1-V2 | Illumina Mi-Seq |
| Urbaniak-2016[14] | BC | 16S rRNA | V6 | Illumina Mi-Seq |
| Kim-2021[15] | BC | 16S rRNA | V1-V3 | Illumina Miseq |
| Chiba-2020[16] | BC | 16S rRNA | V4 | Illumina MiSeq |
| Luo-2023[17] | BC | 16S rRNA gene | 16S rRNA | GridION x5 |
| Meng-2018[18] | BC | 16S rRNA | V1-V2 | Illumina HiSeq |
| Hadzega-2021[19] | BC | Transcriptomic RNA-seq |  |  |
| Zhou-2019[20] | OC | 16S rRNA | V3-V4 | Illumina MiSeq |
| Wang-2020[21] | OC | 16S rRNA | V3-V4 | Illumina MiSeq |
| Wang-2023[22] | OC | 2bRAD sequencing |  | Illumina Nova PE150 |
| Hawkins-2022[23] | EC | 16S rRNA | V1-V3 | Ion Torrent |
| Lu-2021[24] | EC | 16S rRNA | V3-V4 | Illumina HiSeq |
| Wang-2022a [25] | EC | 16S rRNA | V3-V4 | Illumina MiSeq |
| Li-2021[26] | EC | 16S rRNA | V3-V4 | Illumina MiSeq |
| Walther-António-2016[27] | EC | 16S rDNA | V3-V5 | Illumina MiSeq |
| Walsh-2019[28] | EC | 16S rRNA | V3-V5 | Illumina MiSeq |
| Wang-2022b [29] | CC | 16S rDNA | V4 | Illumina MiSeq |

# Supplementary Figure


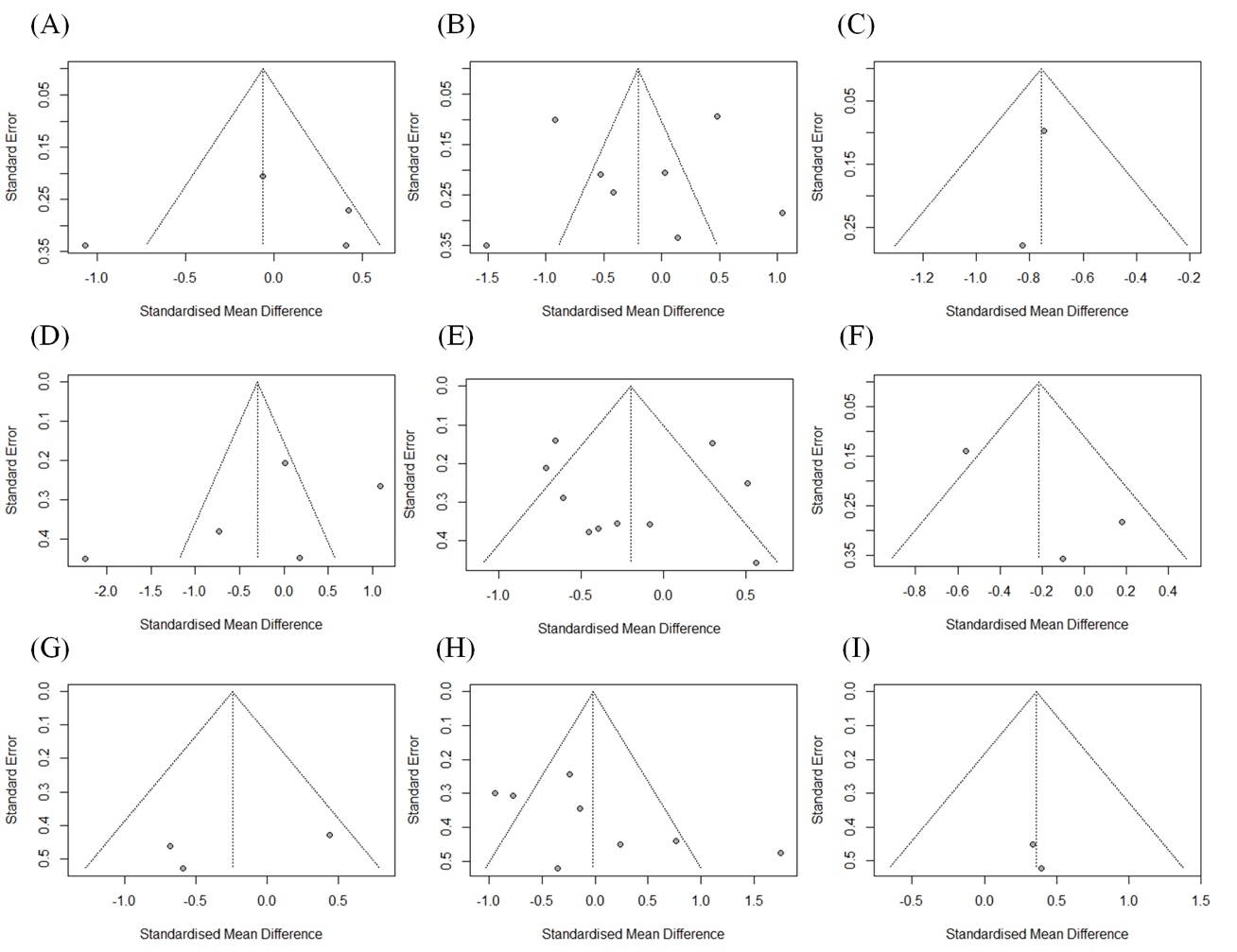


**Figure S1.**  Funnel plots of α-diversity: **(A-C)** tumour tissue versus normal adjacent tissue: **(A)** Chao1; **(B)** Shannon index; **(C)** Simpson index; **(D-F)** tumour tissue versus healthy tissue: **(D)** Chao1; **(E)** Shannon index; **(F)** Simpson index; **(G-I)** tumour tissue versus benign tissue: **(G)** Chao1; **(H)** Shannon index; **(I)** Simpson index.
